# Dispersion of Entropy Perturbations Transporting through an Industrial Gas Turbine Combustor

**DOI:** 10.1007/s10494-017-9854-6

**Published:** 2017-09-21

**Authors:** Yu Xia, Ignacio Duran, Aimee S. Morgans, Xingsi Han

**Affiliations:** 1 Department of Mechanical Engineering, Imperial College London, London, UK; 2 Reaction Engines Ltd., Culham Science Centre, Abingdon, UK; 3 College of Energy and Power Engineering, Nanjing University of Aeronautics and Astronautics, Nanjing, People’s Republic of China

**Keywords:** Combustion noise, Entropy transport, Advective dispersion, Low Mach number LES, ReactingFOAM

## Abstract

In the context of combustion noise and combustion instabilities, the transport of entropy perturbations through highly simplified turbulent flows has received much recent attention. This work performs the first systematic study into the transport of entropy perturbations through a realistic gas turbine combustor flow-field, exhibiting large-scale hydrodynamic flow features in the form of swirl, separation, recirculation zones and vortex cores, these being ubiquitous in real combustor flows. The reacting flow-field is simulated using low Mach number large eddy simulations, with simulations validated by comparison to available experimental data. A generic artificial entropy source, impulsive in time and spatially localized at the flame-front location, is injected. The conservation equation describing entropy transport is simulated, superimposed on the underlying flow-field simulation. It is found that the transport of entropy perturbations is dominated by advection, with both thermal diffusion and viscous production being negligible. It is furthermore found that both the mean flow-field and the large-scale unsteady flow features contribute significantly to advective dispersion — neither can be neglected. The time-variation of entropy perturbation amplitude at combustor exit is well-modelled by a Gaussian profile, whose dispersion exceeds that corresponding to a fully-developed pipe mean flow profile roughly by a factor of three. Finally, despite the attenuation in entropy perturbation amplitude caused by advective dispersion, sufficient entropy perturbation strength is likely to remain at combustor exit for entropy noise to make a meaningful contribution at low frequencies.

## Introduction

Entropy perturbations (or “entropy waves”) are temperature fluctuations generated by unsteady combustion heat release, and are sometimes termed “hot spots”. In gas turbine combustors they play an important role in generating combustion noise [1–6]. Upon being accelerated, as happens predominantly through the combustor chamber exit and the first turbine stage, but also through subsequent turbine stages, entropy perturbations generate “entropy noise” (also known as the “indirect combustion noise”). This constitutes both downstream and upstream propagating acoustic waves [2], with the downstream travelling waves eventually appearing at the turbine outlet [7, 8], which for aero-engines contributes to the total exhaust noise level. The upstream travelling waves, created mainly by the acceleration of entropy perturbations in the first turbine stator row, will propagate back to the flame region, where they may contribute to further flame heat release rate perturbations [9] and affect the propensity to damaging combustion instabilities (also known as “thermoacoustic instabilities”) [10, 11].

The strengths of both acoustic waves induced by entropy noise are directly proportional to the amplitude of entropy perturbations reaching the inlet of the first turbine stage [1], i.e., the combustor chamber exit plane. Many low order analytical models for entropy noise have been developed, e.g., [2, 12–15]. Analytical, experimental and numerical analyses have suggested that for typical gas turbine accelerations (from low subsonic flow to approximately sonic flow), entropy noise may greatly exceed the “direct combustion noise” generated by flame volume expansion. However, a recent analysis suggested that direct combustion noise in fact dominates [16] and so whether entropy noise is the dominant combustion noise source remains an open question.

In order to accurately estimate the entropy noise, the existing low order models all require knowledge of the entropy perturbation amplitude at combustor exit. Thus far, models generally neglect the effect of entropy transport through the combustor chamber, even though numerical simulations suggest that strong entropy perturbations may reach the first turbine stage [17]. The first models of entropy wave transport were by Sattelmayer [18], with Morgans et al. [19] then using turbulent channel flow simulations to show that advective dispersion causes loss of entropy wave strength, but that significant entropy wave amplitude appears likely to remain at the combustor chamber exit. A recent numerical and experimental investigation into the pipe flow in a small-scale rig has been performed by Giusti et al. [20], suggesting that the entropy wave propagation could be described by a frequency response. However, the highly turbulent reacting flow-field in a real industrial gas turbine combustor is quite different to a fully-developed channel flow; flow swirl, separation, recirculation zone and vortex core all act as large-scale hydrodynamic flow features with the potential to further disperse the entropy perturbation strength during its transport process.

Previous large eddy simulations (LES) [21–23] and experiments [24] of combustor reacting flow-fields have shown that the main large-scale flow features, such as flow separation and recirculation zones, are the same whether the flame is fully-premixed [22], partially-premixed [23], or non-premixed [21, 24]. The mean and fluctuating velocity fields are both qualitatively and quantitatively very similar in all cases. It is therefore highly relevant to investigate the effect of these large-scale flow features on the transport of entropy perturbations within a realistic combustor flow-field.

The current work performs a numerical study of the transport of entropy perturbations through a full-scale industrial gas turbine combustor, which contains a highly turbulent reacting flow-field. For the first time, the effects of real combustor hydrodynamic flow features, such as swirl, separation, recirculation, vortex shedding and vortex cores, on the dispersive attenuation of entropy perturbation strength are investigated. The combustor flow-field is numerically simulated using “low Mach number” LES, with the entropy perturbations created at the mean flame front by introducing an artificial time-impulsive “entropy source term”. The subsequent transport of entropy fluctuations within the flow-field is then simulated using a superimposed entropy transport equation, derived directly from the energy conservation equation. This accounts for advection, thermal diffusion and viscous production, and allows the contribution of each of these terms to the entropy transport to be compared. The resulting entropy flux strength at combustor exit as predicted for the fully time-varying combustor flow-field is compared to that predicted using a “frozen” time-averaged flow-field, in order to evaluate the role of unsteady flow structures as compared to mean flow features. Finally, this exit entropy flux strength is compared to that predicted by a turbulent fully-developed pipe mean flow profile, this having the advantage of an analytical form with no simulations required.

The present investigation is relevant to any combustor whose flame generates entropy fluctuations. Recent work for highly simplified (1-D planar and zero Mach number) flames has suggested that entropy disturbances are only generated in the presence of equivalence ratio fluctuations [25, 26]. This paper investigates the transport of entropy perturbations in realistic combustor flow-fields, and does not address the issue of entropy disturbance generation itself. The remainder of the paper is organized as follows: the governing equations of entropy transport are derived in Section 2. The numerical methods and the validation of LES results are presented in Section 3. The simulation results for entropy transport within different flow-fields are presented in Section 4. Conclusions are drawn in the final section.

## Equations Governing Entropy Perturbation Transport

For a compressible viscous fluid flow without external forces, the mass and momentum conservation leads to the Navier-Stokes equations [27]:

1a $$ \frac{\text{D}\rho}{\text{D}t} + \rho \pmb{\nabla} \cdot \boldsymbol{u} =0,  $$

1b $$ \rho \frac{\text{D} \boldsymbol{u}}{\text{D} t} = -\pmb{\nabla} p + \frac{\partial \tau_{\textit{ij}}}{\partial x_{j}}\boldsymbol{e}_{i},  $$

where *ρ* is the fluid density, ***u*** is the velocity vector, *p* is the pressure and *τ*_*ij*_ denotes the viscous stress tensor. The symbol D/D*t* = *∂*/*∂* *t* + ***u*** ⋅∇ represents the material derivative, and ***e***_*i*_ denotes a unit vector in coordinate *i*. For a perfect gas, the gas law gives *p* = *ρ* *R*_*g*_ *T*, with *T* the flow temperature and *R*_*g*_ = *c*_*p*_ − *c*_*v*_ the gas constant, where *c*_*v*_ and *c*_*p*_ are the specific heat capacity at constant volume and pressure, respectively. The conservation of energy then gives the energy equation:

2 $$  \rho \frac{\text{D}}{\text{D}t}\left( e + \frac{1}{2} \boldsymbol{u}^{2} \right) = - \pmb{\nabla} \cdot (p \boldsymbol{u}) + \dot{q} + \pmb{\nabla} \cdot (k \pmb{\nabla} T) + \frac{\partial (\tau_{\textit{ij}} u_{i})}{\partial x_{j}},  $$

where *e* = *c*_*v*_ *T* is the internal energy per unit mass, $\dot {q}$ is the heat addition rate per unit volume, and *k* denotes the thermal conductivity. The specific enthalpy *h* = *c*_*p*_ *T* = *e* + *p*/*ρ* is then defined and combined with Eqs. (1b) and (2), resulting in the enthalpy conservation equation:

3 $$  \rho \frac{\text{D} h}{\text{D} t} = \frac{\text{D} p}{\text{D} t} + \dot{q} + \pmb{\nabla} \cdot (k \pmb{\nabla} T) + \tau_{\textit{ij}} \frac{\partial u_{i}}{\partial x_{j}}.  $$

Since enthalpy *h* also satisfies the thermodynamic relation: d *h* = *T* *d* *s* + *d* *p*/*ρ*, with *s* the specific entropy, the conservation equation for entropy within a fluid flow is then given as [19]:

4 $$  \rho T \frac{\text{D}s}{\text{D}t} = \dot{q} + \pmb{\nabla} \cdot (k \pmb{\nabla} T) + \tau_{\textit{ij}} \frac{\partial u_{i}}{\partial x_{j}},  $$

where ∇⋅ (*k*∇*T*) denotes the thermal gradients which causes diffusion of entropy, while *τ*_*ij*_(*∂* *u*_*i*_/*∂* *x*_*j*_) represents the frictional heating rate that leads to the viscous production of entropy. For entropy transport *downstream of the flame*, the heat addition rate $\dot {q}$ becomes zero; both the thermal conductivity *k* and the dynamic viscosity *μ* (hidden within *τ*_*ij*_) are assumed constant for simplicity.

For small perturbations, a fluid variable can be decomposed into the sum of a mean $\overline {()}$ and fluctuating ()^*′*^ component. Applying this to the definition of entropy, $s = c_{v} {\ln }(p/\rho ^{\gamma })$ (with *γ* = *c*_*p*_/*c*_*v*_ the ratio of specific heat capacities), gives: $s' = c_{v} p^{\prime }/\overline {p} - c_{p} \rho ' / \overline {\rho }$, which is then combined with the ideal gas relation *p* = *ρ* *R*_*g*_ *T* and leads to [5]:

5 $$  \breve{s}^{\prime} = \frac{s^{\prime}}{c_{p}} = \frac{T^{\prime}}{\overline{T}} - \frac{\gamma - 1}{\gamma} \frac{p^{\prime}}{\overline{p}},  $$

where $\breve {s}^{\prime }$ is the non-dimensional entropy fluctuation. The reduced temperature fluctuation ($T^{\prime }/\overline {T}$) is found to be an order of magnitude larger than the pressure fluctuation ($p^{\prime }/\overline {p}$) [5], which then gives $T^{\prime } \approx \overline {T} s^{\prime } / c_{p} = \overline {T} \breve {s}^{\prime }$. It is then combined with Eq. 4 and performs linearisation, resulting in the linearised governing equation for the reduced entropy perturbation $\breve {s}^{\prime }$:

6 $$  \underbrace{\frac{\partial \breve{s}^{\prime} }{\partial t} + \boldsymbol{u} \cdot \pmb{\nabla} \breve{s}^{\prime}}_{\textit{\normalsize{A}}} = \underbrace{ \frac{k}{\rho c_{p}} \frac{\partial^{2} \breve{s}^{\prime}}{\partial x_{i} \partial x_{i}}}_{\textit{\normalsize{D}}} + \underbrace{ \frac{1}{\rho c_{p} \overline{T}} \left( \tau '_{\textit{ij}} \frac{ \partial \overline{u}_{i}}{\partial x_{j}} + \overline{\tau}_{\textit{ij}} \frac{ \partial u_{i}^{\prime}}{\partial x_{j}} \right)}_{\textit{\normalsize{P}}},  $$

where on the left-hand side the linearised entropy advection term is denoted *A*, and on the right-hand side the linearised thermal diffusion and viscous production terms are denoted *D* and *P*, respectively. It is assumed that the entropy fluctuations $\breve {s}^{\prime }$ (and their associated temperature and density fluctuations) have negligible effect on the continuity (Eq. 1a) and momentum (Eq. 1b) equations, commonly known as the Boussinesq approximation. The linearised entropy transport equation, Eq. 6, can then be decoupled from the Navier-Stokes equations and individually solved.

This work seeks an assessment of the relative importance of the individual terms, *A*, *D* and *P*, of the entropy transport equation, Eq. 6, before studying the entropy transport process in a realistic combustor flowfield.

## Large Eddy Simulation of a Turbulent Reacting Flow-field

**Fig. 1:**
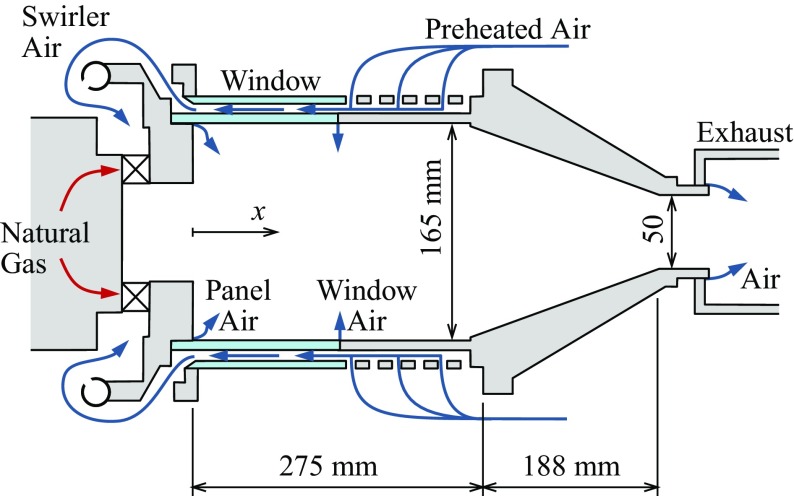
Sketch of the adapted Siemens SGT-100 gas turbine combustor that is studied

The test combustor considered in this work is an adapted Siemens SGT-100 gas turbine combustor [28–30] (see Fig. 1) – the same combustor considered in the previous LES study of Bulat et al. [31]. It consists of a radial swirler entry and a 0.5-metre-long combustion chamber (with a square straight section followed by a circular contraction section), followed by a short exit pipe. Its turbulent reacting flow-field is numerically simulated using “low Mach number” LES. The LES flow conditions match those in the experiments [28, 29]: gaseous methane (CH _4_) fuel is injected at temperature 305 K and technically-premixed with the preheated air at 682 K in the swirler entry of the combustor (see Fig. 1), reaching a global equivalence ratio of 0.60. The upstream operating pressure is 3 bar, with the bulk flow Reynolds number ranging between 18,400 – 120,000 and the bulk Mach number between 0.02 and 0.1. Reacting flow LES are performed using the open-source CFD toolbox, OpenFOAM [32] (version 2.3.0), with the low Mach number LES solver ReactingFOAM, adapted slightly to include an improved model for the turbulent mixing time. The solver utilises a 2nd-order Crank-Nicolson scheme for time derivatives (e.g., $\frac {\partial }{\partial t}$), and adopts a 2nd-order central difference scheme for the convection and diffusion terms. The convective flow speed rather than the speed of sound now determines the computational time step, making the present low Mach number LES significantly faster than the traditional compressible solvers. A fixed time step of 5 × 10^−6^ s is used, this being small enough to avoid losing simulation accuracy or causing divergence of the flow variables, and yielding results which are converged with time-step size and number of (sub-)iterations of the solver. This time step is relatively large compared to traditional compressible solvers (typical time-step size ∼ 10^−7^ s), and thus offers significant time savings.

The governing equations solved by the LES solver ReactingFOAM are the Favre-filtered Navier-Stokes equations of mass and momentum, plus the species mass fraction and energy conservation equations. To close the conservation equations, the constant Smagorinsky model [33] is used for the sub-grid scale (*sgs*) turbulence modelling, together with the van Driest damping method [34, 35] applied to correctly capture the near-wall sub-grid stress tensor behaviour. The PIMPLE method [36] (merged PISO/SIMPLE algorithms) is employed for pressure correction, this solving the pressure equation and momentum corrector with 3 iterations in each time step, and completing two loops over the entire system of equations in each time step. The “finite rate chemistry” Partially-Stirred Reactor (PaSR) model [37] is used to capture the turbulence-combustion interaction, which splits the reacting flow-field in an arbitrary mesh cell into two domains: the first contains the “fine-scale structures”, where all the species are assumed homogeneously mixed and reacted, acting as a “perfectly stirred reactor”. The existence of these fine structures has been proved by recent direct numerical simulations (DNS) [38, 39]. In the second “surrounding domain”, the non-reacting larger scale flow structures dominate and mix with the finer scale burnt products coming from the first domain. By combining both domains, the entire mesh cell can be treated as a “partially-stirred reactor”. The relative proportions of the two PaSR domains in a mesh cell are defined by the reactive volume fraction, *κ* [40]:

7 $$ Y_{i} = (1 - \kappa){Y^{N}_{i}} + \kappa {Y_{i}^{R}},   $$

where *Y*_*i*_ denotes the mass fraction of an arbitrary species *i*, and the superscripts *N* and *R* denote the non-reacting and reacting domain respectively.

Based on the PaSR model, the filtered chemical reaction rate of the *i*-th species, $\overline {\dot {\omega }}_{i}$, can be scaled by *κ* and modelled as [23, 40]:

8 $$  \overline{\dot{\omega}}_{i} \approx \frac{{C_{1}^{i}} - {C_{0}^{i}}}{\Delta t} = \kappa \dot{\omega}_{i} (\overline{\rho}_{m},\widetilde{T}_{m},\widetilde{Y}_{j}, {C_{1}^{i}}),  $$

where ${C_{1}^{i}}$ denotes the mean concentration of species *i* exiting the mesh cell, and ${C_{0}^{i}}$ is the initial mean concentration of species *i* inside the same cell. Δ*t* is the computational time step, and $\widetilde {()}$ denotes the density-weighted (Favre) filtering, defined as $\widetilde {\psi } = \overline {\rho \psi } / \overline {\rho }$ for an arbitrary variable *ψ*. $\dot {\omega }_{i}$ is the unfiltered laminar Arrhenius chemical reaction rate for species *i*, with $\overline {\rho }_{m}$ the filtered mixture density, $\widetilde {T}_{m}$ the Favre-filtered mixture temperature, and $\widetilde {Y}_{j}$ the Favre-filtered mass fraction of species *j* (with *j* = 1,2,…,*N*_*s*_ and *N*_*s*_ the total number of species). The modelling of $\overline {\dot {\omega }}_{i}$ is then reduced to the modelling of *κ*, which is governed by both turbulent mixing and combustion time-scales [23]:

9 $$  \kappa = \frac{\tau_{c}}{\tau_{c} + \tau_{m}},  $$

where *τ*_*c*_ denotes the combustion time, and *τ*_*m*_ is the turbulent mixing time. In order to determine the combustion time *τ*_*c*_, an appropriate chemical reaction mechanism is required [23]. In the present work, a reduced 4-step methane/air reaction mechanism is used, involving 7 intermediate species [41].

The only adaptation made to the default PaSR model is the model for the turbulent mixing time, *τ*_*m*_. The original model [40] using the effective viscosity and sub-grid scale dissipation rate was replaced with a newly-developed mixing model [42, 43], which defined the turbulent mixing time as:

10 $$  \tau_{m} = C_{m} \sqrt{\tau_{\Delta} \cdot \tau_{K}},  $$

where $\tau _{\Delta } = {\Delta } / \sqrt {2k_{\textit {sgs}}/3}$ denotes the sub-grid time-scale, with Δ the mesh cell size and *k*_*sgs*_ the sub-grid kinetic energy. $\tau _{K} = \sqrt {\nu / \epsilon _{\textit {sgs}}}$ is called Kolmogorov time-scale, with *𝜖*_*sgs*_ the sub-grid scale dissipation rate and *ν* the laminar kinematic viscosity. The mixing constant *C*_*m*_ varies with flame structure and is set to 0.8 in order to accurately capture the flame behaviour in the target combustor. This mixing model has been validated by simulations of high Reynolds number, moderate Damk$\ddot {\text {o}}$hler number turbulent flames [42, 43], and was used by Bulat et al. in their recent LES of this same combustor [31].

**Fig. 2:**
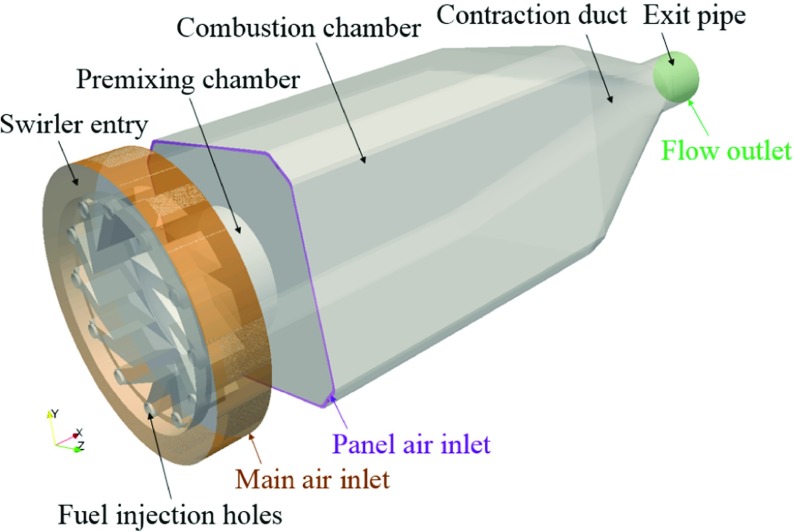
The computational domain, containing 7.0 million structured mesh cells

The computational domain (see Fig. 2) matches that used in Ref. [31]. It consists of a radial swirler entry and a premixing chamber, followed by a dump expansion into the main combustion chamber, where the large-scale complex flow structures (e.g., swirl, recirculation and vortex core) are observed. The main domain inlet is at the swirler entry, including the main air inlet and fuel injection holes. The air inflow velocity at swirler entry is 4.99 m ⋅*s* ^−1^, with the total mass inflow rate of CH _4_ being 6.24 g ⋅*s* ^−1^. The panel air inlet corresponds to a small amount of air entering through the front edges of the combustion chamber, with an inflow velocity of 5.43 m ⋅*s* ^−1^. All the air and fuel inflow velocities are prescribed as uniform. The domain outlet corresponds to the combustor exit plane, with zero velocity gradient and no backward flow. All the solid boundaries are defined as adiabatic non-slip walls, without any heat losses. The semi-logarithm wall-function [44] is applied to all the solid wall boundaries. The whole domain is split into multiple blocks, and each block is meshed with structured cells, resulting in a total of 7.0 million structured mesh cells in the domain [31]. The mesh cell size ranges between 0.281 – 4.16 mm in the axial (x) direction, 0.086 – 5.24 mm in the vertical (y) direction, and 0.083 – 5.33 mm in the transverse (z) direction. Mesh refinement is applied to the swirler entry and the premixing chamber, in order to better capture the fuel/air pre-mixing process [30].

**Fig. 3:**
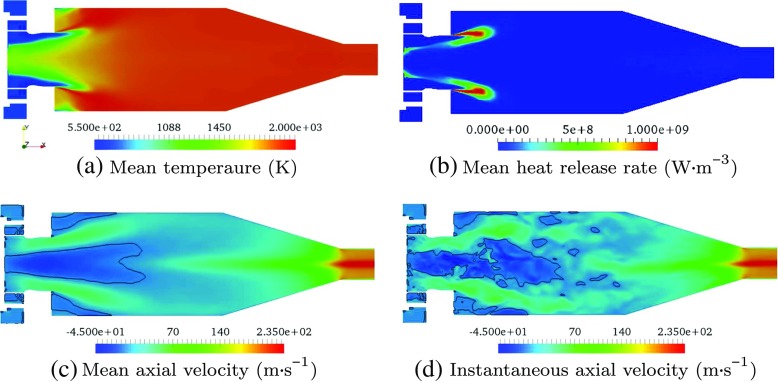
Contours of (**a**) mean flow temperature, (**b**) mean flame heat release rate per unit volume, (**c**) mean flow axial velocity and (**d**) instantaneous flow axial velocity, all simulated by the low Mach number LES on a symmetry plane of *z* = 0. Black curves in (**c**) and (**d**) represent the iso-surfaces of zero axial velocity

**Fig. 4:**
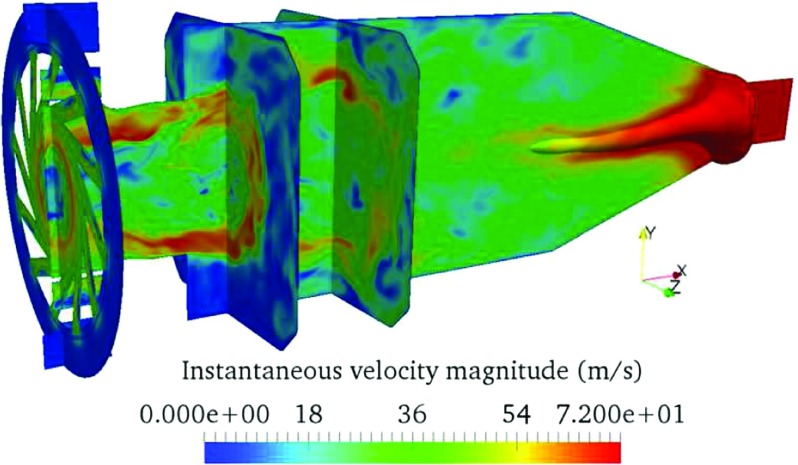
A 3-D contour of instantaneous flow velocity magnitude, with the Central Vortex Core (CVC) marked by the iso-surface of static pressure 310580 Pa, stretching along the combustion chamber centreline from the outlet to the middle part of the chamber

The flow-fields simulated by LES are shown in Figs. 3 and 4. The mean flow temperature and mean heat release rate fields show that the flame region is relatively compact and stabilised close to the combustion chamber entrance. The mean and instantaneous axial flow velocities reveal a main flow separation/recirculation zone along the chamber centreline which extends to the upstream chamber entrance. This zone acts to stabilise the flame front. The non-uniform velocity profile at the exit pipe outlet (see Fig. 3c and d) is due to a coherent flow structure called Central Vortex Core (CVC) [45]. As shown in Fig. 4, this CVC stretches as a 3-D iso-surface of low pressure [46] along the chamber centreline from the outlet to the middle part of the chamber, resulting in a high flow velocity at the centreline but much lower velocities on the two sides, and thus the non-uniform exit velocity profile. The same phenomena have been observed in previous LES [30, 31] and experiments [29].

It is noted that the unsteady reacting flow-field exhibits large-scale flow features such as swirl and vortex shedding, which are not present in the mean flow-field. These flow features are intrinsically unsteady. The unsteady flow-field is clearly dominated by these time-varying hydrodynamic features, unlike the channel flow simulations [19] where the unsteadiness is turbulence dominated.

**Fig. 5:**
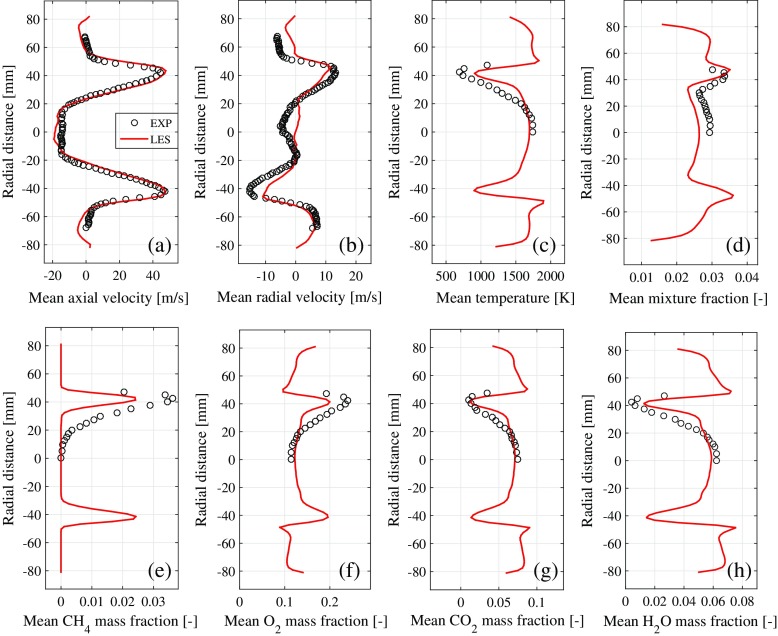
Vertical profiles of mean flow variables predicted by LES (solid line) and measured by experiments [30] (circle), all at an axial location of *x* = 0.043 m, with the origin *x* = 0 located at the combustion chamber entrance

**Fig. 6:**
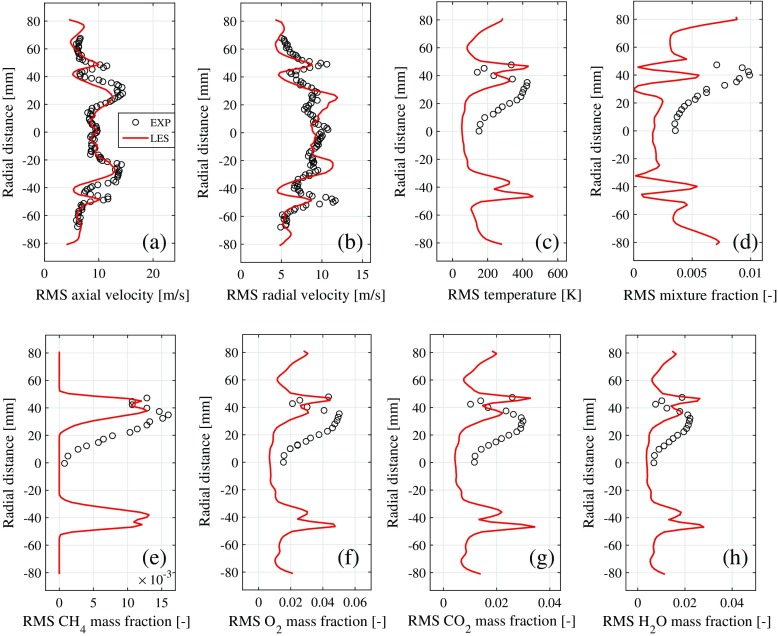
Vertical profiles of fluctuating (RMS) flow variables predicted by LES (solid line) and measured by experiments [30] (circle), all at an axial location of *x* = 0.043 m, with the origin *x* = 0 located at the combustion chamber entrance

The LES results are validated by comparison to available experimental data [30]. Vertical profiles for the mean flow and fluctuating (RMS) flow variables are compared in Figs. 5 and 6 respectively. The match between LES and measurements is generally good, other than some small discrepancies in the RMS profiles (e.g., Fig. 6d). The present LES is thus a sufficiently accurate representation of the turbulent reacting flow-field for the purposes of investigating entropy transport.

## Entropy Perturbation Transport within Combustor Flow-fields

**Fig. 7:**
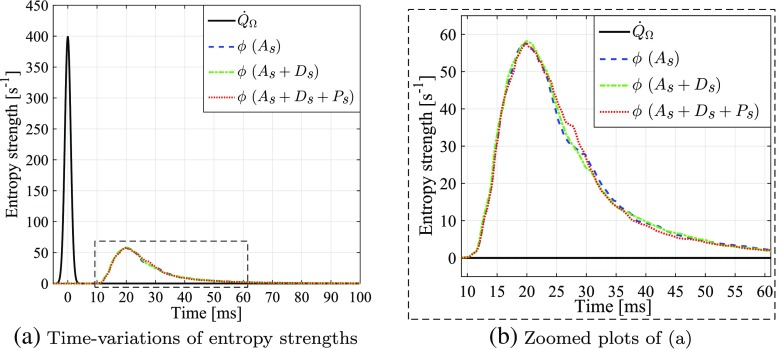
**a** Time-variations of the volume-integrated entropy source term, $\dot {Q}_{\Omega }$ (solid line), and the exiting entropy perturbation fluxes, *ϕ*, after full entropy transport “ *A*_*s*_ + *D*_*s*_ + *P*_*s*_” (dashed line), transport without production “ *A*_*s*_ + *D*_*s*_” (dash-dotted line), and purely advective transport “ *A*_*s*_” (dotted line); **b** Zoomed plots of (**a**) within times *t* = 9 – 61 ms and entropy flux strengths *ϕ* = 0 – 60 s ^−1^

The transport of entropy perturbations within the combustor flow-field can be simulated by solving the governing equation, Eq. 6, superimposed on the underlying flow-field LES. This work does not address the issue of entropy generation, only its transport. Therefore, a generic artificial “entropy source term” [47] is applied, which injects highly localised entropy perturbations into the combustor chamber. In order to introduce entropy perturbations with a sufficiently high bandwidth to allow behaviour across a range of frequencies to be studied, a time-impulsive entropy source term, $\dot {Q}_{\textit {\normalsize {s}}}$, is injected, defined by a sharp Gaussian shape function in time, which approximately mimics a time delta-function. This injection is highly localised spatially, occurring only on the mean flame heat release rate field – see Fig. 3b. This mean flame front location approximately coincides with the location at which entropy could in practice be generated by an appropriate flame. The entropy source term, $\dot {Q}_{\textit {\normalsize {s}}}$, is then expressed as:

11 $$ \dot{Q}_{\textit{\normalsize{s}}}(x,y,z,t)= \frac{\overline{\dot{Q}}_{h} (x,y,z)}{{\iiint_{\Omega}} \overline{\dot{Q}}_{h}(x,y,z) \, \text{d}{V}} \cdot G_{\textit{in}}(t), \ \text{where} \ G_{\textit{in}}(t) = \frac{1}{\sqrt{\pi} {\Delta} \tau_{1}} \exp \left[-\left( \frac{t}{\Delta \tau_{1}} \right)^{2} \right],   $$

where Ω denotes the computational domain and *V* denotes the total domain volume. $\overline {\dot {Q}}_{h} / ({\iiint _{\Omega }} \overline {\dot {Q}}_{h}\, \text {d}{V})$ thus denotes the normalised mean heat release rate spatial field, which gives the mean flame front for entropy injection. The temporal evolution of $\dot {Q}_{\textit {\normalsize {s}}}$ is defined by the Gaussian shape function, *G*_*in*_(*t*), where Δ*τ*_1_ represents the “dispersion time” and has a small value of 1.414 ms, to give a highly time-impulsive signal (see Fig. 7a). *G*_*in*_ is also equal to the spatially-integrated entropy source, ${\iiint _{\Omega }} \dot {Q}_{\textit {\normalsize {s}}} \, \text {d}{V}$, which is also denoted $\dot {Q}_{\Omega }$.

By adding $\dot {Q}_{\textit {\normalsize {s}}}$ as an artificially imposed entropy source on the right-hand side of the entropy transport equation, Eq. 6, we obtain:

12 $$  \underbrace{\frac{\partial S }{\partial t} + \textit{\textbf{u}} \cdot \pmb{\nabla} S}_{\textit{\normalsize{A}}_{\textit{\normalsize{s}}}} = \underbrace{ \frac{k}{\rho c_{p}} \frac{\partial^{2} S}{\partial x_{i} \partial x_{i}}}_{\textit{\normalsize{D}}_{\textit{\normalsize{s}}}} + \underbrace{ \frac{1}{\rho c_{p} \overline{T} {V}} \left( \tau '_{\textit{ij}} \frac{ \partial \overline{u}_{i}}{\partial x_{j}} + \overline{\tau}_{\textit{ij}} \frac{ \partial u_{i}^{\prime}}{\partial x_{j}} \right)}_{\textit{\normalsize{P}}_{\textit{\normalsize{s}}}} + \ \dot{Q}_{\textit{\normalsize{s}}},  $$

where $S=\breve {s^{\prime }}/{V}$ denotes the volume concentration of the normalised entropy perturbation, $\breve {s^{\prime }}$. The advection, diffusion and production of entropy concentration are defined as *A*_*s*_ = *A*/*V*, *D*_*s*_ = *D*/*V* and *P*_*s*_ = *P*/*V*, respectively. Equation 12 is the “forced entropy transport equation”. It is decoupled from the Navier-Stokes equations and is implemented in a superimposed manner within the ReactingFOAM LES solver. In solving for it, a first-order implicit Euler scheme for its time derivatives (e.g., *∂* *S*/*∂* *t*) is used, and a second-order central difference scheme for its spatial discretisation (e.g., ∇*S*) is applied.

### Terms needed to capture the transport of entropy

In order to fully capture the transport of entropy perturbations, the relative importance of each individual term ( *A*_*s*_, *D*_*s*_, *P*_*s*_) in Eq. 12 must be accurately assessed, especially for the thermal diffusion ( *D*_*s*_) and viscous production ( *P*_*s*_) terms. The effects of the large-scale resolved flow structures and the sub-grid scale unresolved turbulent eddies both need to be evaluated.

Before solving Eq. 12 using LES, the non-dimensional form of the linearised entropy transport equation, Eq. 6, is derived. The coordinate *x*, time *t*, flow speed *u*, temperature *T* and shear stress tensor *τ*_*ij*_ are normalised (denoted $\breve {()}$) by the characteristic length *L*, bulk velocity *U*, bulk temperature *T*_*b*_ and dynamic viscosity *μ* as:

13 $$  \breve{x} = \frac{x}{L}, \ \ \ \breve{t} = \frac{tU}{L}, \ \ \ \breve{u} = \frac{u}{U}, \ \ \ \breve{T} = \frac{T}{T_{b}}, \ \ \ \breve{\tau}_{\textit{ij}} = \frac{\tau_{\textit{ij}}L}{\mu U},  $$

the non-dimensional entropy transport equation then becomes:

14 $$  \frac{\partial \breve{s}^{\prime}}{\partial \breve{t}} + \breve{u}_{i} \frac{\partial \breve{s}^{\prime}}{\partial \breve{x}_{i}} = \frac{1}{\textit{Re} \ \textit{Pr}} \frac{{\partial}^{2} \breve{s}^{\prime}}{\partial \breve{x}^{2}_{i}} + \frac{(\gamma -1) M^{2}}{\textit{Re} \ \breve{T}} \left( \breve{\tau}^{\prime}_{\textit{ij}} \frac{\partial \overline{\breve{u}}_{i}}{\partial \breve{x}_{j}} + \overline{\breve{\tau}}_{\textit{ij}} \frac{\partial \breve{u}^{\prime}_{i}}{\partial \breve{x}_{j}} \right),  $$

where *Re* = *ρ* *U* *L*/*μ* denotes the bulk flow Reynolds number, and *M* = *U*/*c* denotes the bulk flow Mach number, with *c* the speed of sound. For the low Mach number ( *M* << 1), large Reynolds number ( *Re* >> 10,000) flows typical of industrial gas turbine combustors, the diffusion and production terms on the right-hand side of Eq. 14 become negligible compared to the advection term on the left-hand side, leading to a “purely advective” entropy transport equation:

15 $$  \frac{\partial \breve{s}^{\prime}}{\partial \breve{t}} + \breve{u}_{i} \frac{\partial \breve{s}^{\prime}}{\partial \breve{x}_{i}} = 0.  $$

Equation 15 indicates that any change in the entropy perturbation strength is primarily caused by the “advective dispersion” of the velocity field, with diffusion and production of entropy having only minor effects. This assumption was applied in previous studies (e.g., [19, 48]), and will be confirmed by LES in this work.

In order to validate above assumption, Eq. 12 is numerically solved using LES by considering both resolved and sub-grid scale effects. Firstly, the thermal diffusion term in Eq. 12 is expressed as:

16 $$  \textit{\normalsize{D}}_{\textit{\normalsize{s}}} = \alpha_{E} \frac{\partial^{2} S}{\partial x_{i} \partial x_{i}}, \ \ \text{where}\ \ \alpha_{\textit{E}} = \frac{\nu}{\textit{Pr}} + \frac{\nu_{\textit{sgs}}}{\textit{Pr}_{\textit{sgs}}},  $$

where the coefficient *k*/(*ρ* *C*_*p*_) in the original diffusion term is replaced by the “effective thermal diffusivity”, *α*_*E*_, and *Pr* = *c*_*p*_ *μ*/*k* denotes the Prandtl number. The subscript “ *sgs*” denotes the contributions from the sub-grid scale turbulent eddies, e.g., *ν*_*sgs*_ the turbulent kinematic viscosity and *Pr*_*sgs*_ the turbulent Prandtl number, both of which are properly defined in OpenFOAM [32].

Secondly, the viscous production term in Eq. 12 is now given as:

17 $$  \textit{\normalsize{P}}_{\textit{\normalsize{s}}} = \frac{1}{\rho c_{p} \overline{T}{V}} \left[ \left( \tau_{\textit{ij}}^{E}\right)' \frac{ \partial \overline{u}_{i}}{\partial x_{j}} + \overline{\tau_{\textit{ij}}^{E}} \frac{ \partial u_{i}^{\prime}}{\partial x_{j}} \right], \ \ \text{where} \ \ \tau^{E}_{\textit{ij}} = \frac{\mu + \mu_{\textit{sgs}}}{2} \left( \frac{\partial u_{i}}{\partial x_{j}} + \frac{\partial u_{j}}{\partial x_{i}} \right),  $$

where *μ* and *μ*_*sgs*_ are the laminar and turbulent dynamic viscosity, respectively.

Three types of entropy transport cases are then simulated in order to capture the contributions of the different terms: (i) full entropy advection, diffusion and production, denoted “ *A*_*s*_ + *D*_*s*_ + *P*_*s*_”, (ii) entropy advection plus diffusion, denoted “ *A*_*s*_ + *D*_*s*_”, in which only the production term is neglected, and (iii) pure entropy advection, denoted “ *A*_*s*_”, in which the diffusion and production terms are both neglected. All three cases apply the same entropy source term $\dot {Q}_{\textit {\normalsize {s}}}$, and use the same unsteady combustor flow-field (see Fig. 3d).

**Fig. 8:**
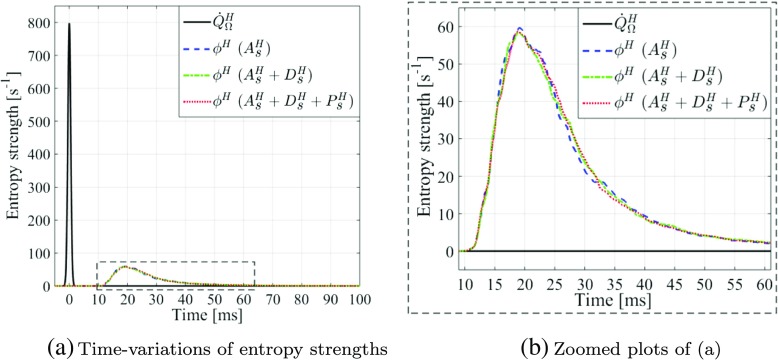
**a** Time-variations of volume-integrated entropy source term, $\dot {Q}^{H}_{\Omega }$, with its dispersion time Δ*τ* halved and peak amplitude doubled (solid line), and the corresponding entropy fluxes at exit, *ϕ* ^*H*^, after full entropy transport “ *A* *s* *H* + *D* *s* *H* + *P* *s* *H*” (dashed line), transport without production “ *A* *s* *H* + *D* *s* *H*” (dash-dotted line), and purely advective transport “ *A* *s* *H*” (dotted line); **b** Zoomed plots of (**a**) within times *t* = 9 – 61 ms and entropy flux strengths *ϕ* ^*H*^ = 0 – 60 s ^−1^

In order to compare the transport of entropy perturbations, the volumetric flux (denoted *ϕ*) of entropy concentration at the combustor exit is defined as:

18 $$  \phi = {\iiint_{\Omega}} \left( \textit{\textbf{u}} \cdot \pmb{\nabla} S \right) \text{d}{V} = {\iint_{\partial{\Omega}}} (\boldsymbol{u} \cdot \textit{\textbf{n}}) S \text{d}\mathit{\mathbb{A}},  $$

where *∂*Ω denotes the surface of the domain Ω, $\mathbb {A}$ denotes the total area of the surface, and vector n denotes the outflow normal direction of the surface. The time-variations of *ϕ* as calculated using three types of entropy transport are shown in Fig. 7. The exit entropy profiles predicted by all three entropy transport cases are almost identical. Their amplitudes are much smaller than that of the entropy source (see Fig. 7a). As shown in Fig. 7b, the effective thermal diffusion term, *D*_*s*_, slightly smooths the time-distribution of *ϕ*, while the viscous production term, *P*_*s*_, marginally increases the magnitude of *ϕ* within times *t* = 25 − 30 ms. Overall, both the diffusion and production terms have negligible effects on the entropy transport, suggesting that subsequent studies in this paper need only account for the advective transport term, *A*_*s*_. A further study of the entropy source term shows that even with the size of the applied entropy impulse doubled (corresponding to Δ*τ* being halved), the same conclusions can still be drawn, as shown in Fig. 8.

As a final note on these entropy transport equation terms, the relative influence of the entropy production term, *P*_*s*_, will depend on the magnitude of the flame-generated entropy waves, which is not included in this study. It is however expected that the flame-generated entropy is orders of magnitude larger than the turbulence-generated entropy. Since the current study does not focus on the generation of entropy waves, but on their advection through the combustion chamber, production terms are neglected in the remainder of this study (i.e., *P*_*s*_ = 0).

The diffusion term of Eq. 12 can also be neglected (i.e., *D*_*s*_ = 0), even within such a highly-turbulent reacting flow-field. This can be done because the sub-grid scale turbulence is negligible when compared to the resolved large-scale turbulent structures of the flow, as the LES is well-resolved. This allows us to apply a simplified purely advective entropy equation to study entropy transport in the reminder of the paper:

19 $$ \underbrace{\frac{\partial S }{\partial t} + \boldsymbol{u} \cdot \pmb{\nabla} S}_{\textit{\normalsize{A}}_{\textit{\normalsize{s}}}} = \dot{Q}_{\textit{\normalsize{s}}}.   $$

### Dispersion of entropy transport within combustor flow-fields

The entropy transport equation, in the form of Eq. 19, is now used to investigate some important features of entropy transport. In order to address the issue of whether entropy advective dispersion is dominated by the mean flow profile, or whether unsteady flow features also play an important role, the effect of the “background” combustor flow-field is investigated. The entropy transport equation is firstly simulated, superimposed on the true time-varying flow-field, combining both time-averaged and unsteady flow features. It is then simulated, superimposed on the “frozen” time-averaged flow-field, in which only the mean flow can play a role. In both cases, the same entropy source term, defined in Eq. 11, is applied.

#### Entropy perturbation transport in a time-varying flow-field

**Fig. 9:**
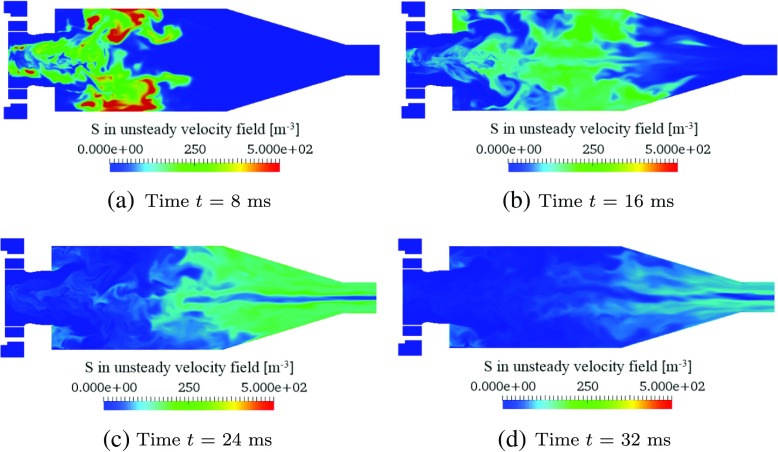
Contours of reduced entropy concentration, *S*, on a symmetry plane of *z* = 0, at four time instants after the impulsive entropy source injection into the time-varying combustor flow-field

The transport of entropy perturbations within the time-varying combustor flow-field is simulated, by superimposing Eq. 19 on the time-varying flow-field from the LES. The resulting spatial distributions of entropy concentration, *S*, at four sequential time instants after the impulsive source injection are shown in Fig. 9. The transport of entropy perturbations is visibly affected by the large-scale unsteady flow features. The central flow separation/recirculation zone has an effect, with entropy transport occurring more quickly towards the side walls, and a small amount of entropy initially pulled upstream and trapped in the central zone.

**Fig. 10:**
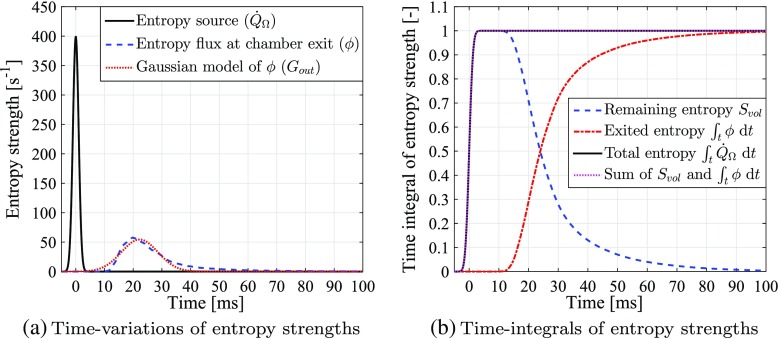
Transport of entropy perturbations within the time-varying combustor flow-field: **a** time-variations of volume-integrated entropy source ($\dot {Q}_{\Omega }$, solid line), exiting entropy concentration flux ( *ϕ*, dashed line), and Gaussian-approximation of *ϕ* ( *G*_*out*_, dotted line); **b** the time-integrated remaining entropy in the combustor ( *S*_*vol*_, dashed line), exited entropy from the combustor (${\int }_{t} \phi \text {d}t$, dash-dotted line), and initially injected entropy into the combustor (${\int }_{t} \dot {Q}_{\Omega } \text {d}t$, solid line). The sum of *S*_*vol*_ and ${\int }_{t} \phi \text {d}t$ is indicated by a dotted line

The time-variation of entropy flux at combustor exit, *ϕ*, is shown in Fig. 10a. The distribution of *ϕ* has a much lower peak amplitude and more spread out time-profile compared to the entropy source impulse, due to the advective dispersion arising from the advective transport. A more detailed analysis can be performed by spatially-integrating the entropy transport equation, Eq. 19, over the domain volume *V*. This gives:

20 $$ \frac{\partial }{\partial t} \underbrace{\left( {\iiint_{\Omega}} S \text{d}{V} \right)}_{\textit{\normalsize{S}}_{\textit{\small{vol}}}} + \underbrace{ {\iint_{\partial {\Omega}}} (\boldsymbol{u} \cdot \textit{\textbf{n}}) S \text{d} \mathbb{A}}_{{\phi}} = \underbrace{{\iiint_{\Omega}} \dot{Q}_{\textit{\normalsize{s}}} \ \text{d}{V}}_{{\dot{Q}_{\Omega}}},  $$

where the first term on the left-hand side can be denoted *∂* *S*_*vol*_/*∂* *t*, with $S_{\textit {vol}} = {\iiint _{\Omega }} S \text {d}{V}$ denoting the amount of entropy remaining in the combustor. The second term is the exiting entropy flux, *ϕ*, defined in Eq. 18. By energy conservation, the sum of *∂* *S*_*vol*_/*∂* *t* and *ϕ* must equal the right-hand side term, ${\iiint _{\Omega }} \dot {Q}_{\textit {\normalsize {s}}} \text {d}{V}$ (denoted $\dot {Q}_{\Omega }$), the total amount of entropy injected by the source term. The time-integrals of *∂* *S*_*vol*_/*∂* *t*, *ϕ* and $\dot {Q}_{\Omega }$ thus satisfy:

21 $$  S_{\textit{vol}} + {\int}_{t} \phi \text{d}t = {\int}_{t} \dot{Q}_{\Omega} \text{d}t.  $$

The first two terms are plotted in Fig. 10b. The entropy flux, *ϕ*, starts to exit the combustor chamber at around *t* = 10 ms, at which point the entropy remaining in the chamber, *S*_*vol*_, starts to drop. The exit rate of *ϕ* is fast for *t* = 10 – 30 ms, but slows down after *t* = 30 ms, resulting in a total of 100 ms for all the entropy to leave the domain. The sum of *S*_*vol*_ and ${\int }_{t} \phi \text {d}t$ always equals the total amount of injected entropy, ${\int }_{t} \dot {Q}_{\Omega }\text {d}t$, obeying the energy conservation law.

In a similar manner as for channel flow advective dispersion (e.g., [19]), the time-variation of *ϕ* can be approximated by a Gaussian shape function, *G*_*out*_:

22 $$  G_{\textit{out}}(t)=\frac{1}{\sqrt{\pi} {\Delta} \tau_{2}} \exp\left[\frac{-(t-\tau_{2})^{2}}{\Delta {\tau_{2}^{2}}}\right],  $$

where *τ*_2_ and Δ*τ*_2_ are the mean delay and dispersion times of *G*_*out*_, respectively, and the amplitude is given by $1/(\sqrt {\pi } {\Delta } \tau _{2})$. Using a non-linear least-squares fitting method [49], *τ*_2_ and Δ*τ*_2_ are calculated to be 22.2 ms and 10.44 ms respectively. The increased dispersion time compared to the entropy source results in a wider Gaussian distribution and a lower peak amplitude. The resulting Gaussian shape function, *G*_*out*_, generally fits the profile of *ϕ* well, deviating slightly from the real shape which exhibits a longer tail, as shown in Fig. 10a.

#### Entropy perturbation transport in the “time-frozen” mean flow-field

**Fig. 11:**
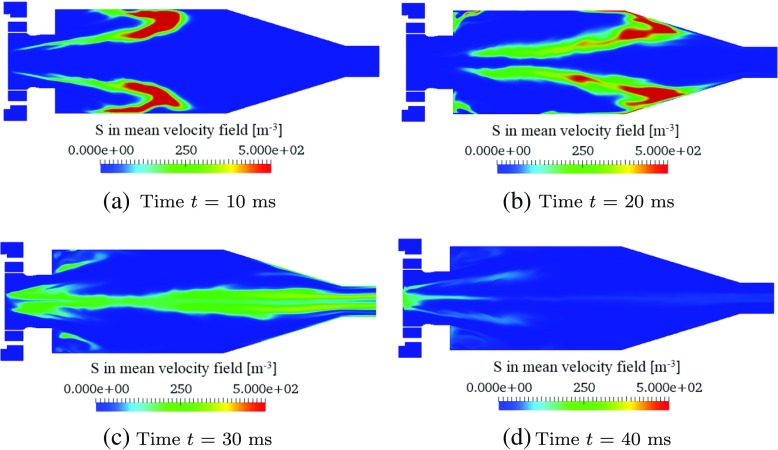
Contours of entropy concentration, *S*, on a symmetry plane of *z* = 0, at four time instants after the impulsive entropy injection into the “‘time-frozen” mean combustor flow-field.

The transport of entropy perturbations superimposed on the “time-frozen” mean combustor flow-field (see Fig. 3c) is simulated by fixing the velocity vector, u, in Eq. 19 to its mean value, $\overline {\textit {\textbf {u}}}$, at each point in space. Four sequential time-snapshots of entropy concentration field after the injection are shown in Fig. 11. The entropy transport is dispersed due to the non-uniform velocity profile. Closer to the combustor side-walls, the flow speeds are higher and the entropy flux reaches the exit plane earlier, while the advection near the centreline is much slower due to the flow recirculation zone. Quite a significant amount of entropy even initially travels back towards the combustor entrance (Fig. 11a–b). As the entropy increasingly exits the domain, the amount of entropy remaining in the combustor decreases (Fig. 11c), with most eventually concentrated in the recirculation zone close to the combustor inlet (Fig. 11d). Although after a sufficiently long time, all of the injected entropy perturbations will leave the combustor, the flow recirculation zone “traps” a small amount of entropy for a long time.

**Fig. 12:**
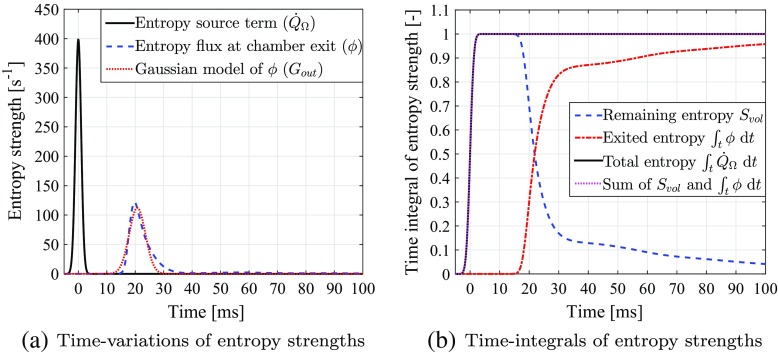
Transport of entropy perturbations within the mean combustor flow-field: **a** time-variations of volume-integrated entropy source ($\dot {Q}_{\Omega }$, solid line), exiting entropy concentration flux ( *ϕ*, dashed line), and Gaussian-approximation of *ϕ* ( *G*_*out*_, dotted line); **b** the time-integrated remaining entropy in the combustor ( *S*_*vol*_, dashed line), exited entropy from the combustor (${\int }_{t} \phi \text {d}t$, dash-dotted line), and initially injected entropy into the combustor (${\int }_{t} \dot {Q}_{\Omega } \text {d}t$, solid line). The sum of *S*_*vol*_ and ${\int }_{t} \phi \text {d}t$ is indicated by a dotted line

The time-evolution of the exiting entropy flux, *ϕ*, is shown in Fig. 12a. Compared to the unsteady flow advection case (see Fig. 10a), the shape of *ϕ*-profile is slightly steeper, with a shorter tail at large times. The time-integrals of *∂* *S*_*vol*_/*∂* *t* and *ϕ* are shown in Fig. 12b. The exiting speed of *ϕ* is much faster at the beginning (*t* = 15 – 30 ms) than for the unsteady flow simulation (see Fig. 10b), but then slows down after *t* = 30 ms, resulting in a much longer total exit time. This delay is due to the “dragging and trapping” effect of the flow recirculation zone, which is the dominant structure in the mean flow-field.

The Gaussian approximation model for *ϕ*, i.e., *G*_*out*_, now takes values *τ*_2_ and Δ*τ*_2_ equal to 20.7 ms and 4.95 ms respectively. This means the time taken for the entropy flux to first reach the combustor exit is almost the same for both time-varying and mean flow-fields, but the mean flow-field has a much weaker dispersion effect overall.

### Entropy transport in an equivalent fully-developed pipe flow

**Fig. 13:**
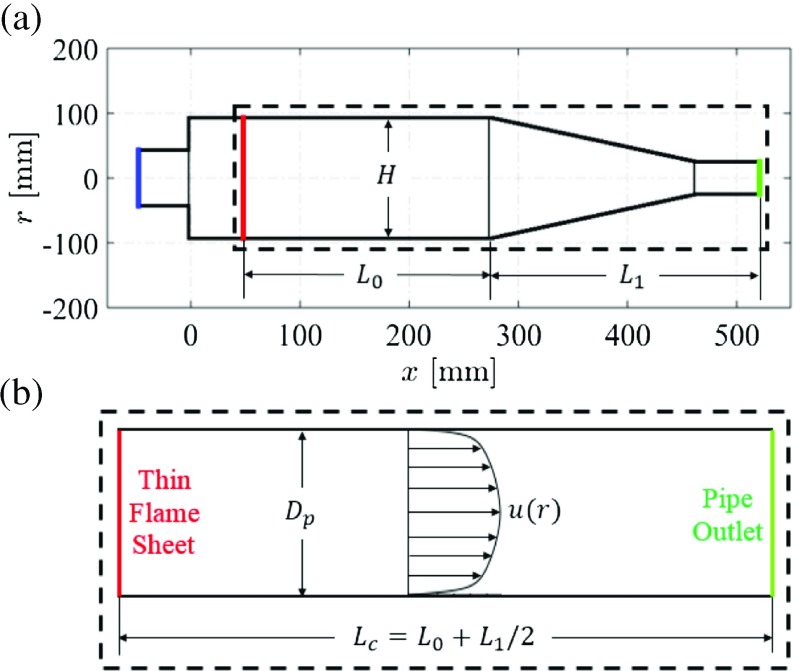
**a** Sketch of the adapted SGT-100 gas turbine combustor (blue line: inlet; red line: thin flame sheet; green line: outlet), with the height of chamber straight section *H* = 0.165 m, the distance between flame and chamber straight-contraction interface *L*_0_ = 0.225 m, and the distance between this interface and combustor exit *L*_1_ = 0.248 m; **b** the equivalent pipe duct geometry, with pipe diameter *D*_*p*_ = *H* = 0.165 m, and pipe length *L*_*c*_ = *L*_0_ + *L*_1_/2 = 0.349 m

Comparison with entropy transport through the mean velocity field of an equivalent pipe geometry is now performed. The motivation for this is to investigate whether an estimate can be obtained for the correct dispersion time, without the need for CFD simulations: the mean turbulent pipe flow profile for a given pipe length and radius are known analytically [47]). As shown in Fig. 13, the original combustor geometry of Fig. 1 is represented as an equivalent straight pipe duct. The pipe diameter, *D*_*p*_, matches the height of the combustor chamber straight section, *H* (0.165 m). The pipe length, *L*_*c*_ = 0.349 m, is calculated using:

23 $$ {L_{c} = L_{0} + \frac{L_{1}}{2},}  $$

where *L*_0_ = 0.225 m denotes the distance from the flame to the chamber straight-contraction interface, and *L*_1_ = 0.248 m the distance from this interface to the chamber exit plane (see Fig. 13a). The axial extent of the flame region is assumed much shorter than the total axial length of the combustor, such that a thin flame “sheet” can be assigned to a single axial location ( *x* = 0.048 m) for calculating *L*_0_. This is the location where the maximum mean heat release rate was measured experimentally [29]. The pipe inlet now corresponds to the thin flame sheet, and the pipe outlet corresponds to the combustor exit plane (see Fig. 13b).

The mean axial flow velocity, $\overline {u}$, within the pipe is 14.97 m ⋅*s* ^−1^, matching the LES-simulated flame-downstream velocity in the test combustor. The axial velocity profile of this pipe flow is time-independent, varying only radially as [47]:

24 $$ u(r) = u_{\textit{max}} - \frac{2.5u_{\textit{max}}}{20}\ln\left( \frac{R}{R-r}\right),   $$

where *r* denotes the radial distance from the centreline, *R* denotes the pipe radius and is equal to *D*_*p*_/2 (0.0825 m), and *u*_*max*_ is the maximum axial velocity, satisfying $u_{\textit {max}} = 1.2 \overline {u}$. The residence time, *τ*_*RES*_, for an entropy perturbation travelling from the flame front (i.e., pipe inlet) to the combustor exit (i.e., pipe outlet) is defined as:

25 $$ \tau_{\textit{RES}}(r) = \frac{L_{c}}{u(r)}.   $$

**Fig. 14:**
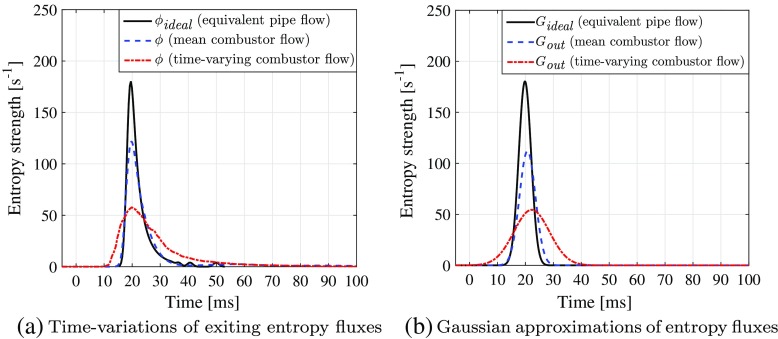
Comparison of entropy perturbation transport within the mean (dashed line) and time-varying (dash-dotted line) combustor flow-fields and the equivalent fully-developed pipe flow (solid line), including (**a**) the time-variations of exiting entropy fluxes and (**b**) the Gaussian approximations of the exiting entropy fluxes

An imposed time-impulsive (delta-function) entropy source, $\dot {Q}_{\textit {ideal}}$, is introduced at the pipe inlet, coinciding with the mean flame front of the real combustor. The time-variation of the responding entropy flux at the pipe outlet, *ϕ*_*ideal*_, is equal to the probability density function (PDF) of the analytical pipe residence time *τ*_*RES*_, i.e., PDF( *τ*_*RES*_) [18]. The predicted maximum amplitude of *ϕ*_*ideal*_ (180.2 s ^−1^) is ∼1.5 times higher than that predicted using mean combustor flow-field (119.8 s ^−1^), and more than three times higher than that for the time-varying combustor flow-field (57.7 s ^−1^) (see Fig. 14a). Thus the time-varying combustor flow appears to attenuate the peak amplitude of the “flame-to-outlet” entropy relation by a factor of roughly three compared to the equivalent fully-developed pipe flow. The time-evolution of *ϕ*_*ideal*_ can also be well-estimated by a Gaussian shape function *G*_*ideal*_ [47] (see Fig. 14b), with its mean delay time calculated to be 19.8 ms. The dispersion time of *G*_*ideal*_ is equal to 3.10 ms, which is much smaller than those for the mean (4.95 ms) and time-varying (10.44 ms) combustor flow-fields.

**Fig. 15:**
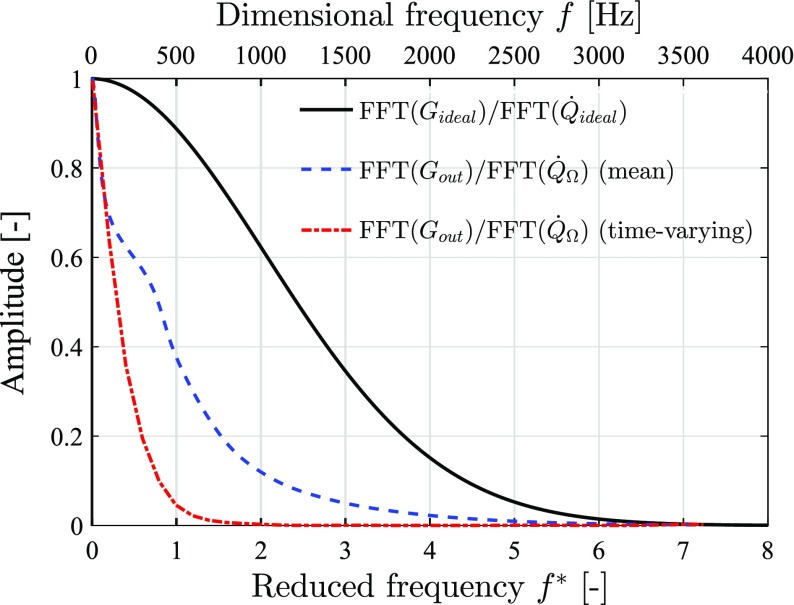
Frequency response amplitudes for entropy transport within the mean (dashed line) and time-varying (dash-dotted line) combustor flows and an equivalent fully-developed pipe flow (solid line), versus both the reduced frequency ( *f* ^∗^) and the dimensional frequency (*f*). *f* ^∗^ and *f* satisfy $f = f^{*} \cdot \overline {u}/L_{c}$, with the characteristic length *L*_*c*_ = 0.349 m. The range of bottom *f* ^∗^-axis ([0,8]) is determined based on a low mean bulk velocity of $\overline {u} =$ 14.97 m ⋅*s* ^−1^, while the top *f*-axis range ([0, 4000] Hz) is obtained based on *f* ^∗^ = [0, 8] and a much higher mean bulk velocity of $\overline {u}$ = 179 m ⋅*s* ^−1^

The frequency responses of entropy perturbation advecting from flame to exit are obtained via fast Fourier transform (FFT) of the relevant signals (see Fig. 15). For the mean and time-varying combustor flow-fields, the volume-integrated entropy source, $\dot {Q}_{\Omega }$, and the Gaussian-fitted exiting entropy fluxes, *G*_*out*_, are used. The small dispersion time Δ*τ*_1_ ensures that $\dot {Q}_{\Omega }$ is close to the ideal impulse $\dot {Q}_{\textit {ideal}}$. The fast Fourier transform ratio, $\text {FFT}({G}_{\textit {out}})/ \text {FFT}(\dot {Q}_{\Omega })$, is then calculated over the frequency domain. For the equivalent fully-developed pipe flow, the delta-function impulsive entropy input, $\dot {Q}_{\textit {ideal}}$, and the subsequent Gaussian fitted output, *G*_*ideal*_, are used, resulting in the fast Fourier transform ratio of $\text {FFT}({G}_{\textit {ideal}})/ \text {FFT}(\dot {Q}_{\textit {ideal}})$.

Figure 15 shows the frequency response amplitudes for entropy transport within three flow-fields, versus both the reduced frequency, *f* ^∗^, and the dimensional frequency, *f*. The reduced frequency *f* ^∗^ is defined as:

26 $$ f^{*} = \frac{f L_{c}}{\overline{u}}.  $$

For mean bulk velocity $\overline {u}= 14.97$ m ⋅*s* ^−1^ and characteristic length *L*_*c*_ = 0.349 m, *f* ^∗^ ranges between 0 and 8 (see Fig. 15), with the corresponding range of *f* being [0, 335] Hz (not shown). The frequency response amplitude for the fully-developed pipe flow has the widest bandwidth, with the mean and time-varying combustor flow-fields giving successively reduced bandwidths. The large-scale unsteady flow features (e.g., swirl) in the time-varying combustor flow thus act to reduce the bandwidth of the entropy transport frequency-response, which is a significant effect that cannot be neglected.

Note that although the geometry of the test combustor is representative of industrial geometries, the test flow speed is lower than that for an industrial operating condition [29, 30]. For industrial operating conditions, a Mach number downstream of combustion of *M*_*d*_ = 0.21 would be typical, corresponding to a mean bulk velocity of $\overline {u}=$ 179 m ⋅*s* ^−1^ (assuming a temperature of *T*_*d*_ = 1800 K and ratio of specific heats *γ* = 1.4). The dimensional frequency range would then extend over [0, 4000] Hz for *f* ^∗^ in the range [0, 8] (see Fig. 15). As can be seen, despite the presence of advective dispersion, significant entropy strength remains at the combustor exit at low frequencies ( ≤ 500 Hz) for all three flow-fields, and thus the entropy noise is likely to be relevant in cases where a large amount of entropy waves are generated.

## Conclusions

For a combustor containing a flame which generates entropy waves as well as an exit flow acceleration, there is the potential for significant entropy noise. It is then essential to know how entropy waves are transported between the flame and the acceleration zone in order to predict the amount of entropy noise. Previous studies on entropy wave transport have been limited to highly simplified geometries: this work has used numerical simulations to study the transport of entropy perturbations within a realistic gas turbine combustor flow-field.

The turbulent reacting flow-field was simulated using a “low Mach number” LES solver called ReactingFOAM. The simulation results accurately matched available experimental data, and captured the main steady and unsteady hydrodynamic flow features common to a wide range of combustor flow-fields, such as swirl, separation, recirculation, a central vortex core, etc. An entropy source term in the form of a time-impulsive distribution was then injected, highly localised in space at a location corresponding to the mean heat release rate field, creating entropy perturbations that transport within the combustor flow-field. It was found that the transport of entropy disturbances was dominated by advection, with both the thermal diffusion and viscous production negligible. The advection of entropy perturbations was then compared for the true time-varying flow-field and a “time-frozen” mean flow-field in the combustor. This revealed that the large-scale unsteady flow features contribute significantly to advective dispersion, and hence to entropy strength attenuation at combustor exit. They cannot be neglected — advective dispersion occurs due to both mean and unsteady flow features. As compared to an equivalent fully-developed pipe flow, whose dispersion can be predicted using a simple analytical expression, dispersion within the time-varying combustor flow is more than tripled due to the large-scale flow features (e.g., swirl, separation, recirculation and CVC). By scaling the reduced frequency response to typical high bulk flow speeds, it is found that despite the presence of advective dispersion, sufficient entropy perturbation strength is likely to remain at combustor exit for low frequency entropy noise to be relevant.
